# Negative effects of density on space use of small mammals differ with the phase of the masting‐induced population cycle

**DOI:** 10.1002/ece3.2513

**Published:** 2016-10-26

**Authors:** Michał Bogdziewicz, Rafał Zwolak, Lauren Redosh, Leszek Rychlik, Elizabeth E. Crone

**Affiliations:** ^1^ Department of Systematic ZoologyFaculty of BiologyAdam Mickiewicz UniversityPoznańPoland; ^2^ Department of BiologyTufts UniversityMedfordMAUSA

**Keywords:** density dependence, home range, mast seeding, population cycles, space use, spatially explicit capture recapture

## Abstract

Home range size generally decreases with increasing population density, but testing how this relationship is influenced by other factors (e.g., food availability, kin structure) is a difficult task. We used spatially explicit capture–recapture models to examine how home range size varies with population density in the yellow‐necked mouse (*Apodemus flavicollis*). The relationship between population density and home range size was studied at two distinct phases of population fluctuations induced by beech (*Fagus sylvatica*) masting: post‐mast peak in abundance (first summer after mast, *n* = 2) and subsequent crash (second summer after mast, *n* = 2). We live‐trapped mice from June to September to avoid the confounding effects of autumn seedfall on home range size. In accordance with general predictions, we found that home range size was negatively associated with population density. However, after controlling for the effect of density, home ranges of mice were larger in post‐mast years than during the crash phase. This indicates a higher spatial overlap among neighbors in post‐mast years. We suggest that the increased spatial overlap is caused by negative density‐dependent dispersal that leads to high relatedness of individuals within population in the peak phase of the cycle.

## 1 Introduction

Space use (home range) allows an animal to access resources necessary to ensure its survival and reproduction (Burt, 1943; Ostfeld, 1990), influences gene flow and interactions with other species, and thus is considered an important feature regulating population dynamics (Adams, 2001; Andreassen, Glorvigen, Rémy, & Ims, 2013; Lambin & Yoccoz, 1998; Schmidt & Ostfeld, 2003). Several factors, including population density, food availability, sex, predation, and kin structure, jointly influence individual spacing behavior (Boutin, 1990; Desy, Batzli, & Liu, 1990; Godsall, Coulson, & Malo, 2014; Kawata, 1990; McLoughlin & Ferguson, 2000; Schoepf, Schmohl, König, Pillay, & Schradin, 2015).

Population density is believed to be the primary determinant of animal space use, with home range area generally decreasing with increasing density (Adams, 2001; Efford, Dawson, Jhala, & Qureshi, 2016). Nonetheless, the relationship between density and spacing behavior is mediated by other factors leading to variation in the spatial overlap among neighboring individuals. For example, higher food availability relaxes the effects of population density on space use (Adams, 2001; Schoepf et al., 2015). Similarly, higher genetic relatedness within population leads to higher spatial overlap among individuals (Le Galliard, Gundersen, Andreassen, & Stenseth, 2006; Pilot, Dąbrowski, Jancewicz, Schtickzelle, & Gliwicz, 2010). At the same time, the increased resource sharing might negatively affect individual reproductive success (Lambin & Krebs, 1993), and spatial overlap among individuals determines the rate of disease transmission (Pedersen & Greives, 2008; Proffitt, White, & Garrott, 2010). Thus, recognizing how density and other factors interact in determining the population spatial structure is crucial to understanding population dynamics (Andreassen et al., 2013). However, separating effects of density from other factors in natural populations is difficult, because different factors covary in space and time (Efford et al., 2016; Schoepf et al., 2015).

Spatially explicit capture–recapture (SECR) models provide a new tool to evaluate temporal or spatial changes in space use in relation to population density (Efford et al., 2016). In SECR models, population density (D) is estimated simultaneously with the spatial scale of detection (σ), a measure of space use (Efford, 2004). Each animal is assumed to occupy a home range center at an unknown location, and each detector (e.g., live trap) is set at know location described by Cartesian coordinates (Borchers & Efford, 2008; Efford, 2004). The detection function describes the increasing probability of detection with decreasing distance between an animal's home range center and the detector (Borchers & Efford, 2008; Efford & Fewster, 2013; Efford et al., 2016). Thus, the spatial scale of detection (σ) increases with the home range, and the parameter σ is a model‐based index of home range size (Efford et al., 2016). Both parameters (D and σ) might vary among populations, and their relationship reflects the degree of overlap between individual home ranges (Efford et al., 2016). This relationship can be parameterized equivalently using *k* that describes the degree of overlap between home ranges ($k=\sigma \sqrt{D}$) (Efford et al., 2016).

**Figure 1:**
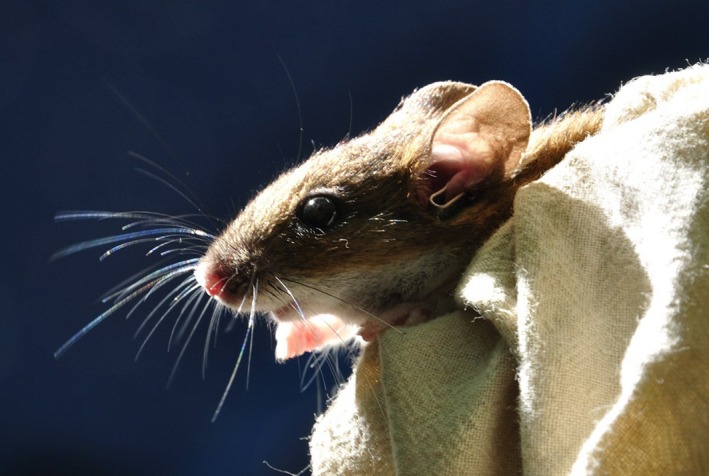
Yellow‐necked mouse (*Apodemus flavicollis*) is a granivorous woodland rodent common in Europe (photo by Stanisław Pagacz)

In this work, we used SECR models to evaluate whether the relationship between population density and small mammals’ space use differs at two distinct phases of the rodent population cycle: postmast peak in abundance (first summer after masting; hereafter FSA) and subsequent crash (second summer after masting; SSA). We used yellow‐necked mouse (*Apodemus flavicollis*; Figure 1) population as a model system. The fluctuations of the studied population are induced by beech (*Fagus sylvatica*) mast seeding (Zwolak, Bogdziewicz, & Rychlik, 2016), that is, the intermittent and synchronized production of seeds (Crone & Rapp, 2014; Kelly, 1994). Strong effects of masting on rodent population dynamics occur in a variety of ecosystems leading to several fold increases in population abundance after mast years (Bogdziewicz, Zwolak, & Crone, 2016; Ostfeld & Keesing, 2000). The general assumption is that the masting‐mediated increase in population density decreases mammals’ home range size (Auger, Meyer, & Jenkins, 2016; Kozakai et al., 2011; Lacher & Mares, 1996; McShea & Schwede, 1993; Stradiotto et al., 2009). However, at the distinct phases of the population cycle generated by masting, other factors (e.g., kin structure) might vary as well, leading to variation in the relationship between rodent density and spatial behavior. Such effects could alter the density–home range area relationship with potential consequences for population dynamics.

Our null hypothesis is that the effects of masting on rodent space use are solely density‐mediated, that is, the relationship between density and home range area does not differ between FSA (i.e., peak of the population cycle) and SSA (subsequent crash phase of the population cycle) years. Alternatively, the relationship could differ between the phases of the population cycle, revealing more complicated effects of masting on rodent populations. The direct influence of beech seed abundance on space use of mice is ruled out, because we sampled rodent populations only during summer, when beech seeds are unavailable (they are produced in the fall and germinate or rot in the spring). Therefore, we are able to use relationships between density and home range use in FSA and SSA years to test whether changes in density are sufficient to explain changes in home range size or whether additional factors need to be invoked. Based on patterns in our data, we discuss additional factors that might be responsible for relationships between density and space use in different phases of mast‐induced population cycles.

## 2 Methods

### 2.1 Natural history

The yellow‐necked mouse is a granivorous woodland rodent that is common in deciduous forests of central and eastern Europe. In beech forests, mice rely on spatially clumped and fluctuating resources (nuts) (Jensen, 1982; Zwolak et al., 2016), and mast seeding of beech causes strong fluctuations in the abundance of mice (Jensen, 1982; Zwolak et al., 2016). Mast of deciduous trees is the main food source of the mouse (>80% of the diet), in both mast and nonmast years (Dróżdż, 1966; Selva, Hobson, Cortés‐Avizanda, Zalewski, & Donázar, 2012). The post‐mast increase in rodent abundance is driven by high overwinter survival and winter breeding (Jensen, 1982; Pucek, Jedrzejewski, Jedrzejewska, & Pucek, 1993). Factors affecting the post‐outbreak crash in rodent numbers are less known, but low food availability, predation, and disease are likely candidates (Pedersen & Greives, 2008; Pucek et al., 1993). Females’ space use is expected to be driven by food availability, and males’ space use by female distribution (Ostfeld, 1990; Stradiotto et al., 2009). Thus, females are expected to maintain smaller and more exclusive territories than males (Ostfeld, 1990; Stradiotto et al., 2009).

### 2.2 Study site

We trapped small mammals in Gorzowska Forest (Map S1, Appendix S1), situated in western Poland. The forest is located in temperate climate zone at an altitude of 60–80 m. Average annual precipitation equals 523 mm, and average annual temperature 8°C. Common tree species include *Fagus sylvatica*,* Quercus* spp., *Pinus sylvestris*, and *Larix decidua*. For the study, we selected eight sites solely occupied by beech trees. Distances among sites averaged 1.6 km (*SD* = 0.8 km, range: 0.4–6 km). More detailed description of study sites can be found in Zwolak et al. (2016).

### 2.3 Small mammal trapping

We trapped small mammals during four years (2010–2013), in four monthly sessions (June–September). We divided the sites into two sets, and sites within each set were trapped simultaneously for five consecutive nights (i.e., 40 960 trap nights in total). At each site, we set up 8 × 8 trapping grids with 10‐m spacing between trap stations. One wooden live trap (“dziekanówka” type, widely used in Poland, size 21 × 8 × 9.5 cm) was placed at each trap station and baited with rolled oats and sunflower seeds. The traps that we used are designed for single catches, but double catches sometimes occurred. We checked traps in the morning (starting at 08:00) and in the evening (starting at 18:00). We identified captured rodents to species, determined their sex, and marked them with uniquely numbered ear tags.

### 2.4 Beech nut production

We determined yearly beech seed production by counting seeds on the ground (Hilton & Packham, 1997). We sampled beech by collecting and counting all seeds in 0.25 m^2^ squares (24 per site in 2009 and 12 per site in 2010–2012) centered on randomly selected trap stations. Each year, we selected the points in a stratified random manner: Each site was divided into four subplots, with six (2009) or three (2010–2012) trap stations per subplot used as sampling points. The sampling was conducted once per year in late October after seeds had fallen in mid‐October.

### 2.5 Spatially explicit capture–recapture models

We estimated the population density (D) by fitting models using the detection function λ (*d*; λ_0_, σ) that describes the decline in cumulative probability of detection λ with increasing distance *d* between an animal home range center and a trap (Borchers & Efford, 2008; Efford et al., 2016). The parameter λ_0_ represents the probability of detecting an individual when a trap is located at its activity center. The parameter σ is the spatial scale of detection that describes the relationship between detection probability and the distance between a trap and an animal activity center, that is, a metric of home range size (Efford et al., 2016). We fitted models using the “secr” package in R (Efford, 2015). We assumed home range centers to follow a uniform Poisson process (for details see, e.g., Borchers & Efford, 2008; Efford & Fewster, 2013). The detection function followed a half‐normal curve. We used models with multicatch traps, but estimates of D and σ are robust to this kind of model misspecification (Efford, Borchers, & Byrom, 2009a). We set the spatial buffer over the grid at 100 m after checking that density estimates did not vary with increased width.

We fitted separate models to data for male and female mice because the SECR models are computationally intense and fitting the global model for the complete dataset was not feasible. In addition, we expected a priori that space use would differ between males and females, with stronger territoriality in female mice (Ostfeld, 1990). For simplicity, we used only morning catches in the analysis (these constituted >98% of total mouse captures). Model parameters (D, λ_0_, and σ) were set to be constant or varying among trapping sessions; λ_0_ also included (global) behavioral difference between initial and subsequent captures (i.e., trap happy or trap shy). We allowed D, λ_0_, and σ to vary independently. Thus, we fitted 12 models for each sex representing all possible combinations of these three parameters. All models also included separate parameters for each site, that is, the most simple, “constant” model included eight estimates of λ_0_, D, and σ (one for each site). Thus, the most complicated model included 48 estimates of each parameter, one for each of 16 trapping sessions at each site. The best model was selected with the Akaike's information criterion corrected for small sample size, AICc (Burnham & Anderson, 2002). We also present Akaike weights (*w*_i_), which can be interpreted as the weight of evidence in favor of a particular model relatively to other considered models (Burnham & Anderson, 2002).

We also explored models with density dependence of sigma differing only between phases of the mast‐induced population cycle (using the *k* re‐parameterization of SECR, see Efford et al., 2016). However, more complicated models with σ and density differing independently with years and sites fitted data far better (according to AICc scores, presumably due to other factors that also differed among sites and years). Therefore, we fitted more complicated models to avoid biased parameter estimates and then tested whether the density versus sigma relationship is affected by masting with generalized linear mixed models. Based on the estimated D_p_ and σ_p_, we calculated session‐specific *k*_*p*_ and used this parameter to calculate *S*_95_ (*S*_95_ = 6π*k* ^2^), which represents an estimate of the number of individuals that occurs at any time within the area of an individual's 95% home range limits (for details and assumptions see Efford et al., 2016).

### 2.6 Generalized linear mixed models

We explored the relationship between SECR‐based estimates of home range size (σ), rodent density, and mast seeding with generalized linear mixed models (GLMMs) implemented in R using “lme4” package (Bates, Maechler, Bolker, & Walker, 2015). In the first model, we tested whether rodent density differs according to masting history and between males and females. Here, we used log‐transformed rodent density (D) as response variable, and mast seeding (FSA vs. SSA), sex, and two‐way interaction as fixed effects. In the second model, we tested whether home ranges differ according to mast history, population density, and sex. Here, we used log‐transformed σ as the response variable, and log‐transformed mouse density, mast seeding, sex, and all their two‐way interactions as fixed effects. In both models, we used study site as a random effect and month as a covariate. We used Gaussian family, identity link models, and tested for statistical significance of fixed factors with Wald Type II test, implemented via the “car” package in R (Fox & Weisberg, 2011).

## 3 Results

Beech produced abundant seed crops in 2009 (mean ± *SD*: 345 ± 80 nuts/m^2^) and 2011 (382 ± 83 nuts/m^2^). In 2010 and 2012, beech failed to produce seeds: No nuts were found on the ground or observed on the tree branches.

For both sexes, density (D) and home range size (σ) varied among trapping sessions (Table 1). In males, model assuming D and σ varying among sessions strongly outperformed all others (ΔAICc ≥ 77.11). In females, the difference between this model and the second best was smaller (ΔAICc = 3.60), but the evidence ratio (*w*_1_/*w*_2_) for the best model versus the second was 6.03. Therefore, we estimated D and σ separately for each trapping session (for parameter estimates see Table 1 in Appendix) and input these estimates into GLMMs.

**Table 1:** Model selection table, identifying the most parsimonious models of density and homer range of a) females and b) males of yellow‐necked mice

Model structure					
λ_0_	*D*	σ	#P	ΔAICc	w_i_
(a) *Females*					
Behavioral response	Session	Session	230	0	0.86
Session + Behavioral response	Session	(.)	230	3.60	0.14
Session + Behavioral response	Session	Session	329	57.71	<0.001
Behavioral response	Session	(.)	131	100.32	<0.001
Session	Session	(.)	222	111.84	<0.001
(b) *Males*					
Behavioral response	Session	Session	242	0	1
Session + Behavioral response	Session	Session	347	77.12	<0.001
Session + Behavioral response	Session	(.)	242	107.72	<0.001
Behavioral response	Session	(.)	137	156.52	<0.001
(.)	Session	Session	234	159.57	<0.001

Only the best five candidate models are shown.

λ_0_, detection probability; D, density; σ, spatial scale of detection (i.e., metric of home range size); (.), constant; session, varying among trapping sessions. The models were ranked according to ΔAICc; #P denotes the number of parameters, and w_i_ can be interpreted as the weight of evidence in favor of model i (Burnham & Anderson, 2002).

**Figure 2:**
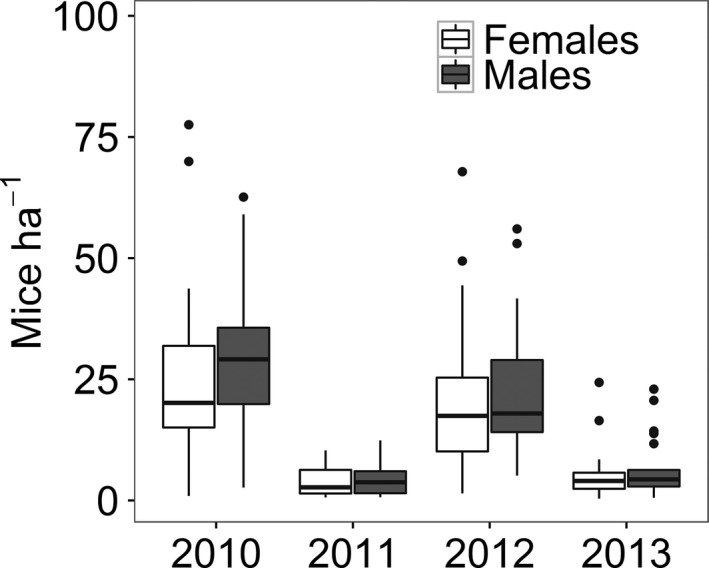
Density of females and males of the yellow‐necked mouse in Gorzowska Forest (W Poland). Monthly (4 months) site‐specific (eight grids) densities are averaged to show differences among years. Beech masting occurred in 2009 and 2011. Density is derived from SECR models that received best AIC support (see Table 1 and 2 for details). Boxes denote 25th, 50th, and 75th percentiles; whiskers represent the lowest and highest datum within the 1.5 interquartile range

Mouse densities were higher in FSA than in SSA years (main effect of “mast,” χ^2^ = 177.19, *p* < .001), and density of males was higher than that of females (the main effect of “sex,” χ^2^ = 5.10, *p* = .02, Figure 2). The effect of mast seeding on rodent density did not differ between sexes (mast × sex interaction, χ^2^ = 0.01, *p* = .90). In the crash phase, the average densities were estimated as 6.37 ± 5.95 (mean ± *SD*) individuals/ha in males and 5.26 ± 5.33 inds/ha in females. In the peak phase, the density increased fourfold: to 27.68 ± 17.61 inds/ha in males and 22.36 ± 15.89 inds/ha in females.

**Figure 3:**
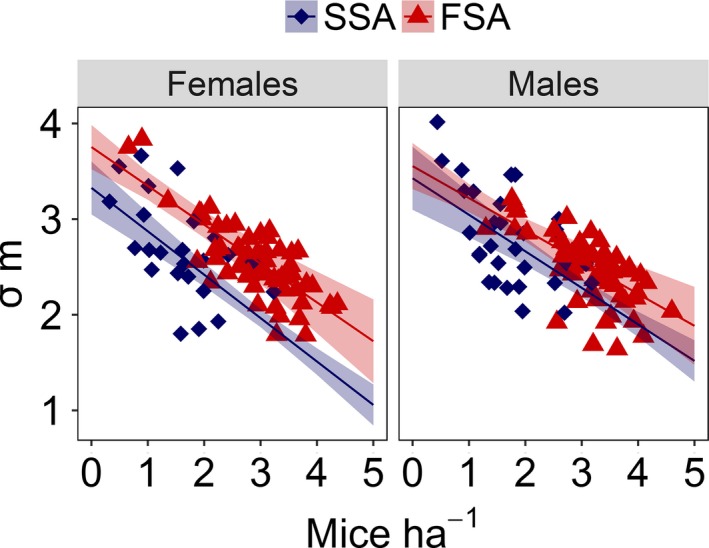
Relationship between density of the yellow‐necked mouse and sigma (σ, model‐derived estimate of home range size) in first summer after masting and second summer after masting years. Dots represent session‐specific estimates of parameters. Note that both axes are on log scale. The log‐log slope of fitted curves equals −0.5, while the difference in intercepts indicates differences in home range overlap (i.e., higher intercept denotes larger home ranges for the same level of density). Trend lines are reported with 95% confidence intervals and are based on predictions from generalized linear mixed model (see 2 section for details)

As expected, home range size declined with density (main effects of density in Table 2, Figure 2). Home range size also differed between phases of the mast‐induced population cycle (the main effects of “mast” in Table 2, Figure 3). After correcting for changes in density, yellow‐necked mice had larger home ranges in FSA than in SSA years. Home range sizes also differed significantly between male and female mice; males had larger home ranges (the main effect of Sex in Table 2, Figure 3). We also observed a significant Mast × Sex interaction (Table 2); home range size of female mice differed more between phases of the mast‐induced population cycle than home range size of males. No other interactions were statistically significant (Table 2).

**Table 2:** Statistical significance of GLMM fixed effects testing the relationship between mast seeding and rodent space use

Fixed effect	χ^2^	*p*
Mast	28.09	<.001
Sex	3.98	.05
Density	148.71	<.001
Mast× sex	4.40	.03
Mast × density	2.69	.07
Sex× density	0.53	.46

The response variable is log‐transformed sigma (i.e., SECR‐derived metric of home range size). Study site was used as random effect. Degrees of freedom for all effects equal 1

The parameter *k* (index of home range overlap, Efford et al., 2016) averaged among sites and months (±*SE*) was 0.54 (±0.01) for females and 0.58 (±0.01) for males in FSA, and 0.30 (±0.02) for females and 0.37 (±0.02) for males in SSA. The parameter *S*_95_ (the number of individuals within the area of one home range, Efford et al., 2016) was 5.93 for females and 6.75 for males in FSA, and 2.04 for females and 3.06 for males in SSA.

## 4 Discussion

Our study shows that density‐mediated effects alone are not sufficient to explain changes in mouse spatial behavior evoked by mast seeding. Past studies have generally concluded that masting reduces space use through increased population density (Auger et al., 2016; Mazurkiewicz & Rajska‐Jurgiel, 1998; Stradiotto et al., 2009). Our results partly support this prediction, in that σ, the index of home range size, declined with increasing density and was generally lower in FSA compared to SSA years. However, for the same level of density, home range sizes were larger in the peak phase of the cycle (FSA) in comparison with the crash phase (SSA). This indicates a higher degree of space overlap between neighboring individuals, as shown by *k* and *S*_95_ estimates (Efford et al., 2016). It is not clear whether mice are more tolerant of overlap in space use in post‐mast years or whether they are constrained to use more space, relative to overall densities. However, it is clear that the effects of mast seeding on space use in mice are more complicated than simple density‐mediated changes in space use.

In order to explain the difference in spatial overlap during different phases of the mast‐induced population cycle, we need to invoke a mechanism that involves something other than the density itself. Relatedness among individuals would be likely to differ between FSA and SSA summers after masting because, for a given density, postmast populations are in the peak phase after growth during the mast event, whereas SSA populations have grown to this density after a low density period. When mouse population density rises and territories are filled, dispersal rates typically decline in rodents (Ims & Andreassen, 2000, 2005; Lambin & Krebs, 1991; Smith & Batzli, 2006; Wolff, 1996). This process leads to higher relatedness of neighboring individuals (Andreassen et al., 2013; Pilot et al., 2010; Sutherland, Spencer, Singleton, & Taylor, 2005; Wolff, 1996). Home range overlap is higher between more closely related individuals (Ims, 1989; Kawata, 1990; Lambin & Krebs, 1993; Le Galliard et al., 2006; Wolff, 1996), probably due to reduced aggression (Kawata, 1990; Lambin & Krebs, 1993). This effect occurs in both sexes, but is stronger in females than in males (Innes et al., 2012; Ishibashi, Saitoh, Abe, & Yoshida, 1997; Le Galliard et al., 2006; Pilot et al., 2010), which is consistent with the stronger effect of masting years on female mice in our study.

The pattern of high spatial overlap among individuals in the peak phase of the population cycle found in our study is similar to that found in rodent population cycles that are not driven by masting, but by a set of intrinsic (e.g., sociality, dispersal) and extrinsic (e.g., predation) factors (Andreassen et al., 2013; Radchuk, Ims, & Andreassen, 2016). In such systems, spatial overlap enhances reproduction at the beginning of the population growth phase, but after a critical point, it triggers population collapse (reviewed in Andreassen et al., 2013). Reproduction is first enhanced because the benefits of sharing space (e.g., protection against infanticide) outpace the costs (e.g., competition for food). In the latter phase, intensified crowding slows down reproduction, and the crash is caused by predation of dominant males, which disrupts social groups and further decreases survival (Andreassen & Gundersen, 2006; Ims & Andreassen, 2000; Odden, Ims, Støen, Swenson, & Andreassen, 2014). Similar sets of intrinsic factors might be responsible for population regulation across a variety of territorial mammals (Odden et al., 2014).

Such processes have not been studied in mast‐induced population cycles, although we know that dispersal rates decline during postmast (peak) years in yellow‐necked mouse (Mazurkiewicz & Rajska‐Jurgiel, 1998) and that reproduction ceases in the peak phase of the cycle (Falls, Falls, & Fryxell, 2007; Fitzgerald, Efford, & Karl, 2004; Mazurkiewicz & Rajska‐Jurgiel, 1998; Pucek et al., 1993; Wolff, 1996). In mast‐generated population cycles, rodent abundance is still growing during early summer after masting, although beech seeds are already depleted (consumed, germinated, or rotten), and the decline begins in late summer or autumn (Falls et al., 2007; Pucek et al., 1993; Zwolak et al., 2016). Our study points that the number of individuals within one home range is 2‐ to 3‐fold higher in FSA than in SSA. This is very likely to affect the competition for resources and disease transmission and, thus, play a role in the population decline. In that context, it might be illuminating to study how dispersal, spatial organization, reproduction, and survival covary across the whole mast‐induced population cycle.

The increase in spatial overlap of home ranges that was found after mast years could be caused by other factors. Although availability of beech seeds was most likely constant across years (because trapping was conducted when this food source was unavailable), availability of other food items could vary. For example, in conifer forests of North America, masting‐mediated increase in density, survival, and reproduction in deer mice (*Peromyscus maniculatus*) was delayed to summer after masting (Lobo & Millar, 2013). Authors suggested that the fir (*Abies lasiocarpa*) masting resulted in population response of invertebrate seed predators that translated into higher prey availability for rodents (Lobo & Millar, 2013). Such an effect could potentially prolong the window of increased food availability after masting into next year summer and affect spatial behavior of mice. Investigating whether masting results in the second‐order pulse in invertebrate numbers could be an interesting avenue for future research.

Spatially explicit capture–recapture models provide an effective tool to separate the effects of density on space use from other factors (Efford et al., 2016) and allowed us to show that the patterns of space use differ between distinct phases of rodent population cycle. One advantage is that our research was based on an extensive dataset that allowed the estimation of population‐wide changes in space use. This scale is usually infeasible in telemetry‐based studies that are necessarily limited to a smaller subsample of individuals. Moreover, live trapping (or analogous methods based on proximity detectors: Efford, Dawson, & Borchers, 2009b; Efford, 2011) is a widely used research method. This wide use means that SECR models can be applied to separate the effects of density from other important biological factors in a wide range of ecological problems, for example, in studies testing the influence of habitat type on space use, in studies on multi‐annual population cycles of voles and lemmings where spacing behavior is likely to be a key component of population regulation (Andreassen et al., 2013; Efford et al., 2016; Wolff, 1996), or to study spacing behavior of pests to inform management policy (Ringler et al., 2014). We hope that our study will encourage future applications of this method.

## Conflict of interest

The authors declare no conflict of interest.
