# Supplementary figures and images for: Negative effects of density on space use of small mammals differ with the phase of the masting‐induced population cycle
Source: Ecol Evol. 2016 Oct 26;6(23):8423–30. doi: 10.1002/ece3.2513 (PMC5167038; doi:10.1002/ece3.2513)

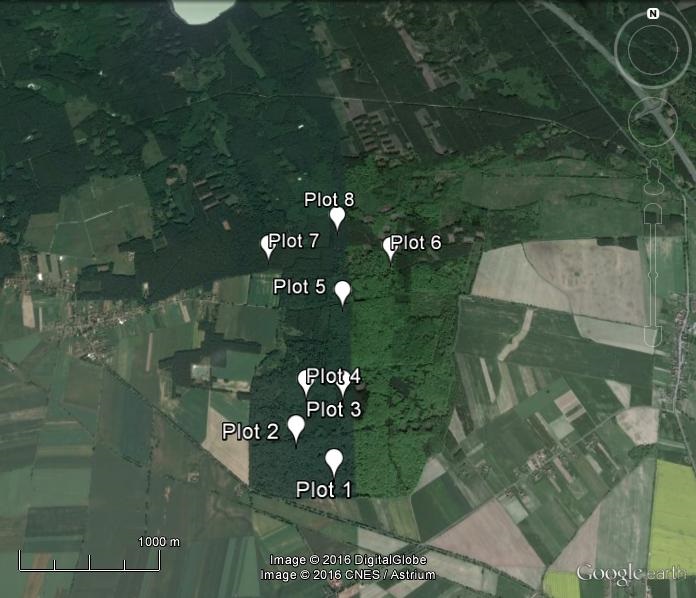

Supplement: Supplementary file 1 [file ECE3-6-8423-s001.jpg]
