# Supplementary material for: Negative effects of density on space use of small mammals differ with the phase of the masting‐induced population cycle
Source: Ecol Evol. 2016 Oct 26;6(23):8423–30. doi: 10.1002/ece3.2513 (PMC5167038; doi:10.1002/ece3.2513)
Supplement: Supplementary file 2 [file ECE3-6-8423-s002.docx]

Table 1S. Number of individuals (Minimum Number Known Alive; MNKA ) of (A) females and (B) males of yellow-necked mice (*Apodemusflavicolis*) live-trapped during the study along with SECR-based model estimates of the density (D) and sigma (σ). Estimates are derived from models that received the best support according to AICc (see the main article for details). Parameter *k* was estimated post-hoc based on D and σ estimates.

A) Females

| Year | Month | Site | MNKA | D | σ | *k* |
| --- | --- | --- | --- | --- | --- | --- |
| 2010 | June | 183 | 32 | 77.53 | 8.28 | 0.72 |
| 2010 | July | 183 | 31 | 69.95 | 7.98 | 0.66 |
| 2010 | August | 183 | 14 | 15.03 | 13.93 | 0.54 |
| 2010 | September | 183 | 5 | 5.79 | 21.45 | 0.51 |
| 2010 | June | 120 | 4 | 0.93 | 42.59 | 0.41 |
| 2010 | July | 120 | 20 | 40.97 | 8.35 | 0.53 |
| 2010 | August | 120 | 14 | 19.31 | 13.64 | 0.59 |
| 2010 | September | 120 | 30 | 33.83 | 14.09 | 0.81 |
| 2010 | July | 144 | 17 | 20.14 | 12.96 | 0.58 |
| 2010 | August | 144 | 27 | 33.15 | 12.36 | 0.71 |
| 2010 | September | 144 | 18 | 15.28 | 16.41 | 0.64 |
| 2010 | June | 169 | 20 | 43.72 | 5.98 | 0.39 |
| 2010 | July | 169 | 26 | 39.05 | 7.08 | 0.44 |
| 2010 | August | 169 | 15 | 14.06 | 12.24 | 0.45 |
| 2010 | September | 169 | 26 | 28.29 | 10.93 | 0.58 |
| 2010 | June | 159 | 6 | 7.28 | 10.38 | 0.28 |
| 2010 | July | 159 | 24 | 30.66 | 11.24 | 0.62 |
| 2010 | August | 159 | 20 | 17.01 | 13.06 | 0.53 |
| 2010 | September | 159 | 23 | 21.1 | 13.01 | 0.59 |
| 2010 | June | 193 | 8 | 15.38 | 11.21 | 0.43 |
| 2010 | July | 193 | 18 | 19.09 | 17.24 | 0.75 |
| 2010 | August | 193 | 13 | 18.08 | 14.14 | 0.60 |
| 2010 | September | 193 | 21 | 27.31 | 14.55 | 0.76 |
| 2010 | June | 195 | 13 | 27.23 | 7.29 | 0.38 |
| 2010 | July | 195 | 25 | 36.94 | 9.59 | 0.58 |
| 2010 | August | 195 | 21 | 26.51 | 10.52 | 0.54 |
| 2010 | September | 195 | 8 | 2.89 | 24.28 | 0.41 |
| 2010 | June | 182 | 9 | 15.08 | 11.28 | 0.43 |
| 2010 | July | 182 | 22 | 21.73 | 16.57 | 0.77 |
| 2010 | August | 182 | 11 | 8.44 | 18.6 | 0.54 |
| 2010 | September | 182 | 10 | 6.09 | 20.13 | 0.49 |
| 2011 | July | 183 | 13 | 10.32 | 13.81 | 0.44 |
| 2011 | September | 183 | 3 | 6.31 | 9.48 | 0.24 |
| 2011 | July | 144 | 2 | 0.63 | 34.87 | 0.28 |
| 2011 | June | 169 | 3 | 1.53 | 21.02 | 0.26 |
| 2011 | July | 159 | 4 | 6.31 | 13.01 | 0.33 |
| 2011 | August | 193 | 1 | 1.79 | 14.55 | 0.19 |
| 2011 | July | 195 | 4 | 3.62 | 11.41 | 0.22 |
| 2011 | July | 182 | 1 | 1.16 | 14.86 | 0.16 |
| 2012 | June | 183 | 26 | 67.84 | 8.15 | 0.67 |
| 2012 | July | 183 | 26 | 44.37 | 10.21 | 0.68 |
| 2012 | August | 183 | 35 | 38.1 | 14.39 | 0.89 |
| 2012 | September | 183 | 11 | 14.72 | 12.28 | 0.47 |
| 2012 | June | 120 | 11 | 12.9 | 12.3 | 0.44 |
| 2012 | July | 120 | 11 | 25.23 | 8 | 0.40 |
| 2012 | August | 120 | 11 | 12.47 | 14.84 | 0.52 |
| 2012 | September | 120 | 16 | 10.01 | 18.63 | 0.59 |
| 2012 | June | 144 | 20 | 21.16 | 14.26 | 0.66 |
| 2012 | July | 144 | 29 | 34.04 | 11.33 | 0.66 |
| 2012 | August | 144 | 22 | 30.14 | 11.01 | 0.60 |
| 2012 | September | 144 | 13 | 12.83 | 14.64 | 0.52 |
| 2012 | June | 169 | 12 | 18.15 | 8.18 | 0.35 |
| 2012 | July | 169 | 13 | 25.7 | 6 | 0.30 |
| 2012 | August | 169 | 20 | 17.95 | 11.64 | 0.49 |
| 2012 | September | 169 | 12 | 5.98 | 21.32 | 0.52 |
| 2012 | June | 159 | 8 | 10.16 | 11.45 | 0.36 |
| 2012 | July | 159 | 22 | 29.71 | 9.55 | 0.52 |
| 2012 | August | 159 | 19 | 11.8 | 19.03 | 0.65 |
| 2012 | September | 159 | 14 | 7.18 | 22.73 | 0.61 |
| 2012 | June | 193 | 11 | 21.1 | 11.3 | 0.52 |
| 2012 | July | 193 | 11 | 19.3 | 11.29 | 0.50 |
| 2012 | August | 193 | 6 | 7.96 | 14.6 | 0.41 |
| 2012 | September | 193 | 9 | 1.45 | 46.31 | 0.56 |
| 2012 | June | 195 | 12 | 16.96 | 9.96 | 0.41 |
| 2012 | July | 195 | 20 | 24.59 | 10.75 | 0.53 |
| 2012 | August | 195 | 33 | 49.41 | 9.97 | 0.70 |
| 2012 | September | 195 | 8 | 8.15 | 13.28 | 0.38 |
| 2012 | June | 182 | 20 | 15.11 | 11.04 | 0.43 |
| 2012 | July | 182 | 6 | 5.53 | 12.9 | 0.30 |
| 2012 | August | 182 | 35 | 22.56 | 17.76 | 0.84 |
| 2012 | September | 182 | 6 | 8.83 | 14.88 | 0.44 |
| 2013 | June | 183 | 5 | 1.75 | 28.36 | 0.38 |
| 2013 | July | 183 | 3 | 4.57 | 11.01 | 0.24 |
| 2013 | August | 183 | 13 | 16.48 | 12.82 | 0.52 |
| 2013 | September | 183 | 11 | 3.61 | 34.17 | 0.65 |
| 2013 | September | 120 | 6 | 1.42 | 39 | 0.46 |
| 2013 | August | 144 | 4 | 4.03 | 14.54 | 0.29 |
| 2013 | July | 144 | 1 | 0.38 | 24.15 | 0.15 |
| 2013 | June | 144 | 2 | 5.73 | 6.37 | 0.15 |
| 2013 | September | 144 | 13 | 24.35 | 9.39 | 0.46 |
| 2013 | August | 169 | 2 | 1.91 | 11.83 | 0.16 |
| 2013 | July | 169 | 2 | 3.88 | 6.06 | 0.12 |
| 2013 | September | 169 | 3 | 2.41 | 14.15 | 0.22 |
| 2013 | July | 159 | 4 | 8.48 | 6.89 | 0.20 |
| 2013 | September | 159 | 4 | 4.17 | 12.58 | 0.26 |
| 2013 | August | 195 | 3 | 3.63 | 11.4 | 0.22 |
| 2013 | September | 195 | 9 | 7.92 | 16.53 | 0.47 |
| 2013 | September | 182 | 7 | 5.16 | 19.63 | 0.45 |

B) Males

| Year | Month | Site | MNKA | D | σ | *k* |
| --- | --- | --- | --- | --- | --- | --- |
| 2010 | June | 183 | 35 | 62.6 | 10.35 | 0.82 |
| 2010 | July | 183 | 21 | 29.12 | 13 | 0.70 |
| 2010 | August | 183 | 22 | 34.69 | 11.81 | 0.70 |
| 2010 | September | 183 | 26 | 20.59 | 12.44 | 0.56 |
| 2010 | July | 120 | 17 | 42.37 | 8.51 | 0.55 |
| 2010 | August | 120 | 18 | 19.21 | 15.26 | 0.67 |
| 2010 | September | 120 | 29 | 36.3 | 13.45 | 0.81 |
| 2010 | July | 144 | 6 | 25.09 | 8.63 | 0.43 |
| 2010 | June | 144 | 17 | 23.59 | 5.41 | 0.26 |
| 2010 | August | 144 | 26 | 31.62 | 10.32 | 0.58 |
| 2010 | September | 144 | 14 | 13.49 | 13.73 | 0.50 |
| 2010 | June | 169 | 20 | 59.03 | 5.9 | 0.45 |
| 2010 | July | 169 | 29 | 51.32 | 8.78 | 0.63 |
| 2010 | August | 169 | 25 | 28.06 | 10.91 | 0.58 |
| 2010 | September | 169 | 29 | 22.62 | 17.58 | 0.84 |
| 2010 | June | 159 | 14 | 36.62 | 5.17 | 0.31 |
| 2010 | July | 159 | 40 | 45.67 | 12.07 | 0.82 |
| 2010 | August | 159 | 22 | 23.48 | 13.65 | 0.66 |
| 2010 | September | 159 | 28 | 29.13 | 13.94 | 0.75 |
| 2010 | June | 195 | 14 | 49.5 | 6.91 | 0.49 |
| 2010 | July | 195 | 22 | 29.56 | 13.3 | 0.72 |
| 2010 | August | 195 | 23 | 28.86 | 14.03 | 0.75 |
| 2010 | September | 195 | 4 | 2.66 | 18.2 | 0.30 |
| 2010 | June | 182 | 4 | 11.92 | 6.83 | 0.24 |
| 2010 | July | 182 | 14 | 32.68 | 7.29 | 0.42 |
| 2010 | August | 182 | 16 | 11.25 | 17.88 | 0.60 |
| 2010 | September | 182 | 9 | 4.82 | 24.81 | 0.54 |
| 2010 | August | 193 | 11 | 14.2 | 14.7 | 0.55 |
| 2010 | July | 193 | 24 | 33.52 | 11.56 | 0.67 |
| 2010 | June | 193 | 12 | 13.39 | 13.65 | 0.50 |
| 2010 | September | 193 | 20 | 34.98 | 10.04 | 0.59 |
| 2011 | July | 183 | 17 | 12.4 | 20.13 | 0.71 |
| 2011 | July | 144 | 6 | 1.39 | 33.51 | 0.40 |
| 2011 | August | 169 | 3 | 1.5 | 26.96 | 0.33 |
| 2011 | June | 169 | 3 | 0.68 | 36.91 | 0.30 |
| 2011 | July | 159 | 7 | 3.75 | 23.56 | 0.46 |
| 2011 | July | 195 | 6 | 4.75 | 31.94 | 0.70 |
| 2011 | September | 195 | 11 | 11.48 | 10.29 | 0.35 |
| 2011 | July | 193 | 2 | 3.58 | 12.69 | 0.24 |
| 2011 | September | 193 | 2 | 6.03 | 7.67 | 0.19 |
| 2012 | June | 183 | 32 | 98.33 | 7.67 | 0.76 |
| 2012 | July | 183 | 25 | 41.67 | 11.67 | 0.75 |
| 2012 | August | 183 | 34 | 56.03 | 11.2 | 0.84 |
| 2012 | September | 183 | 9 | 12.63 | 11.8 | 0.42 |
| 2012 | June | 120 | 14 | 24.11 | 10.67 | 0.52 |
| 2012 | July | 120 | 17 | 18.37 | 15.7 | 0.67 |
| 2012 | August | 120 | 11 | 22.99 | 9.46 | 0.45 |
| 2012 | September | 120 | 13 | 13.16 | 15.84 | 0.57 |
| 2012 | June | 144 | 26 | 39.44 | 8.99 | 0.56 |
| 2012 | July | 144 | 43 | 53.01 | 9.3 | 0.68 |
| 2012 | August | 144 | 26 | 31.59 | 11.06 | 0.62 |
| 2012 | September | 144 | 16 | 16.29 | 12.95 | 0.52 |
| 2012 | June | 169 | 12 | 29.84 | 6.84 | 0.37 |
| 2012 | July | 169 | 14 | 15.78 | 12.12 | 0.48 |
| 2012 | August | 169 | 16 | 12.37 | 16.84 | 0.59 |
| 2012 | September | 169 | 9 | 5.12 | 18.12 | 0.41 |
| 2012 | June | 159 | 12 | 16.37 | 12.16 | 0.49 |
| 2012 | July | 159 | 23 | 28.69 | 11.53 | 0.62 |
| 2012 | August | 159 | 16 | 11.75 | 17.87 | 0.61 |
| 2012 | September | 159 | 10 | 5.35 | 21.81 | 0.50 |
| 2012 | June | 195 | 19 | 27.92 | 11.99 | 0.63 |
| 2012 | July | 195 | 17 | 22.28 | 13.58 | 0.64 |
| 2012 | August | 195 | 29 | 41.68 | 11.69 | 0.75 |
| 2012 | September | 195 | 7 | 6.61 | 17.38 | 0.45 |
| 2012 | June | 182 | 8 | 17.52 | 8.49 | 0.36 |
| 2012 | July | 182 | 10 | 16.15 | 11.31 | 0.45 |
| 2012 | August | 182 | 32 | 26.66 | 16 | 0.83 |
| 2012 | September | 182 | 10 | 5.13 | 23.22 | 0.53 |
| 2012 | August | 193 | 15 | 14.43 | 20.4 | 0.77 |
| 2012 | July | 193 | 17 | 17.02 | 14.3 | 0.59 |
| 2012 | June | 193 | 18 | 19.38 | 13.22 | 0.58 |
| 2012 | September | 193 | 7 | 15.91 | 13.96 | 0.56 |
| 2013 | June | 183 | 3 | 0.55 | 55.49 | 0.41 |
| 2013 | July | 183 | 9 | 14.33 | 12.68 | 0.48 |
| 2013 | August | 183 | 12 | 22.97 | 10.28 | 0.49 |
| 2013 | September | 183 | 7 | 6.14 | 17.31 | 0.43 |
| 2013 | September | 120 | 4 | 5.2 | 14.67 | 0.33 |
| 2013 | August | 144 | 4 | 5.61 | 9.9 | 0.23 |
| 2013 | June | 144 | 4 | 1.94 | 26.79 | 0.37 |
| 2013 | September | 144 | 9 | 5.17 | 31.93 | 0.73 |
| 2013 | August | 169 | 2 | 3.27 | 10.33 | 0.19 |
| 2013 | July | 169 | 2 | 2.88 | 10.4 | 0.18 |
| 2013 | June | 169 | 2 | 3.01 | 15.16 | 0.26 |
| 2013 | September | 169 | 5 | 3.77 | 19.3 | 0.37 |
| 2013 | July | 159 | 3 | 6.29 | 12.13 | 0.30 |
| 2013 | September | 159 | 3 | 4.35 | 9.79 | 0.20 |
| 2013 | August | 195 | 4 | 3.26 | 19.37 | 0.35 |
| 2013 | September | 195 | 13 | 11.71 | 18.04 | 0.62 |
| 2013 | June | 182 | 2 | 2.26 | 13.74 | 0.21 |
| 2013 | September | 182 | 15 | 20.64 | 12.56 | 0.57 |
| 2013 | August | 193 | 2 | 2.28 | 13.96 | 0.21 |
| 2013 | July | 193 | 4 | 13.81 | 7.55 | 0.28 |
| 2013 | September | 193 | 2 | 1.75 | 17.43 | 0.23 |
